# Growing and dividing: how O-GlcNAcylation leads the way

**DOI:** 10.1016/j.jbc.2023.105330

**Published:** 2023-10-12

**Authors:** Harmony Saunders, Wagner B. Dias, Chad Slawson

**Affiliations:** 1 Department of Cancer Biology, University of Kansas Medical Center, Kansas City, Kansas, USA; 2 Federal University of Rio De Janeiro, Rio De Janeiro, Brazil; 3 Department of Biochemistry and Molecular Biology, University of Kansas Medical Center, Kansas City, Kansas, USA

**Keywords:** O-GlcNAc, O-GlcNAc transferase, O-GlcNAcase, cell cycle, mTOR, cyclin, spindle, mini-chromosome complex, nutrient sensing, p53

## Abstract

Cell cycle errors can lead to mutations, chromosomal instability, or death; thus, the precise control of cell cycle progression is essential for viability. The nutrient-sensing posttranslational modification, O-GlcNAc, regulates the cell cycle allowing one central control point directing progression of the cell cycle. O-GlcNAc is a single N-acetylglucosamine sugar modification to intracellular proteins that is dynamically added and removed by O-GlcNAc transferase (OGT) and O-GlcNAcase (OGA), respectively. These enzymes act as a rheostat to fine-tune protein function in response to a plethora of stimuli from nutrients to hormones. O-GlcNAc modulates mitogenic growth signaling, senses nutrient flux through the hexosamine biosynthetic pathway, and coordinates with other nutrient-sensing enzymes to progress cells through Gap phase 1 (G_1_). At the G_1_/S transition, O-GlcNAc modulates checkpoint control, while in S Phase, O-GlcNAcylation coordinates the replication fork. DNA replication errors activate O-GlcNAcylation to control the function of the tumor-suppressor p53 at Gap Phase 2 (G_2_). Finally, in mitosis (M phase), O-GlcNAc controls M phase progression and the organization of the mitotic spindle and midbody. Critical for M phase control is the interplay between OGT and OGA with mitotic kinases. Importantly, disruptions in OGT and OGA activity induce M phase defects and aneuploidy. These data point to an essential role for the O-GlcNAc rheostat in regulating cell division. In this review, we highlight O-GlcNAc nutrient sensing regulating G_1_, O-GlcNAc control of DNA replication and repair, and finally, O-GlcNAc organization of mitotic progression and spindle dynamics.

A fundamental behavior of all cells is to sense the environment and respond accordingly. When nutrients are abundantly paired with appropriate hormonal signals, cell replication can occur. Cell cycle progression is a highly regulated function where close monitoring of the environment is critical, and any environmental alteration can halt the entire process (Fig. 1). Vital to cell progression is O-GlcNAcylation. Simply, O-GlcNAc is the attachment of a single N-acetylglucosamine sugar to serine and/or threonine amino acids in nuclear, cytoplasmic, or mitochondrial proteins (1). Like most posttranslational modifications, O-GlcNAc regulates protein function, stability, localization, or interactions. Importantly, O-GlcNAc is processed by a single O-GlcNAc transferase (OGT), which adds the modification, and a single O-GlcNAcase (OGA), which removes the modification (2). Processing of the modification is sensitive to nutrient and hormonal flux leading to a homeostatic level of O-GlcNAcylation that can rapidly fluctuate in response to changes in OGT and OGA activity. Thus, O-GlcNAcylation acts as a rheostat to fine-tune cellular function.

**Figure 1:**
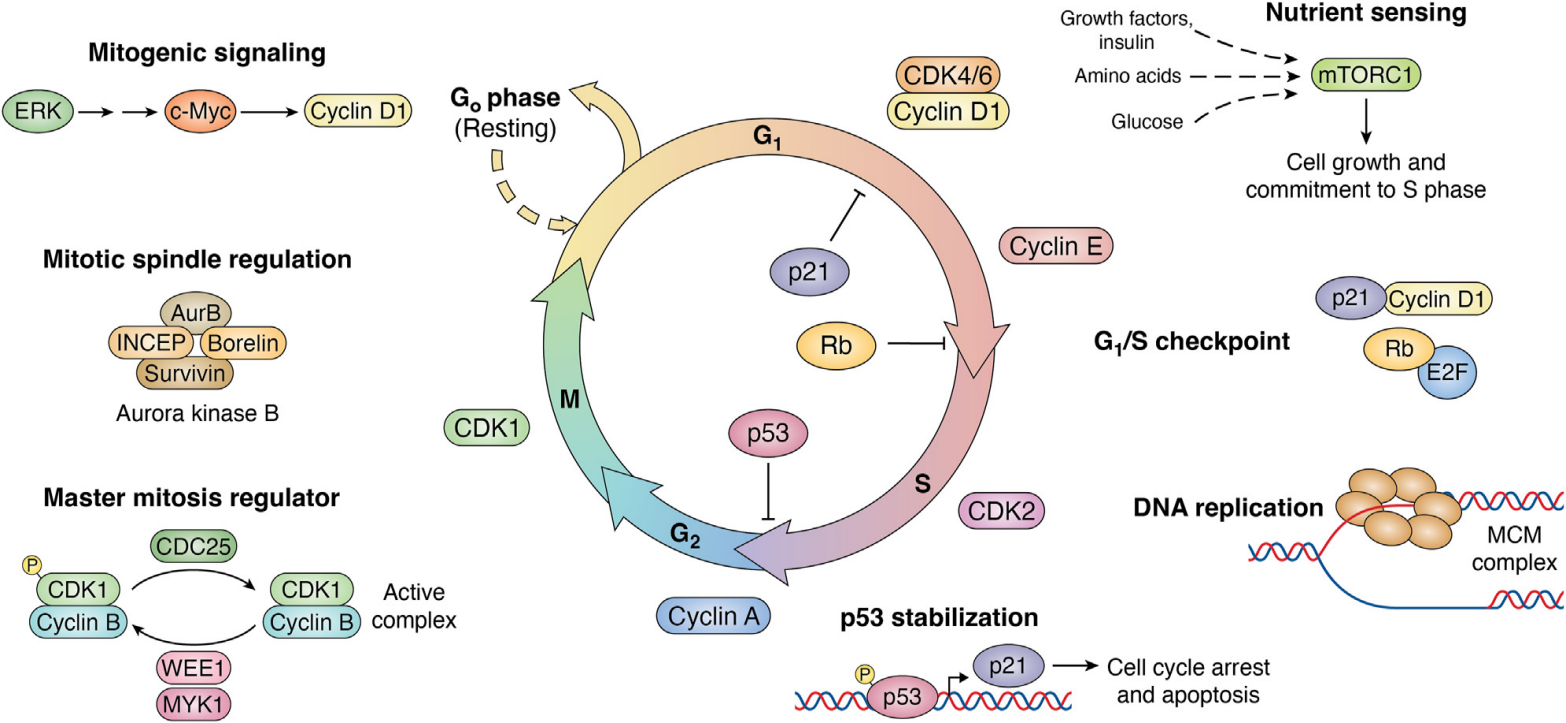
**Overview of the cell cycle.** The cell cycle begins with gap phase 1 (G_1_) followed by DNA synthesis phase (S), Gap phase 2 (G_2_), and division during the mitotic phase (M). Cyclins, cyclin dependent kinases (CDK), and checkpoints regulate cell cycle progression in each phase. Mitogenic (ERK) signaling *via* growth factors, elevated CDK4/6-cyclin D activity, and increased mTORC1 function are essential for G_1_ progression. After disruption of retinoblastoma protein (pRB)–E2F interaction at the G_1_/S checkpoint, S phase utilizes the mini-chromosome complex (MCM) to synthesize DNA. DNA synthesis errors are checked at S and G2, and activation of the transcription factor p53 is essential to arrest the cell cycle by increasing cyclin-dependent kinase inhibitor p21 while errors are fixed. During mitosis, cyclin B bound to CDK1 regulates mitosis *via* phosphorylation, and other mitotic kinases such as the Aurora Kinase B complex directs cell division. ERK, extracellular activated protein kinase; mTORC, mammalian target of rapamycin complex.

The concept of O-GlcNAc as a rheostat is not new to the field of glycobiology, and numerous studies demonstrate that oscillation of O-GlcNAc levels to modify signaling pathways in response to environmental changes (3). Here, having a single enzyme to add or remove the modification allows the cell to harmonize all cellular functions with fluctuating nutrient or growth signals. A single control point, the addition and removal of O-GlcNAc, allows for coordination of all processes across the cell; however, this implies that O-GlcNAc changes could have subtle and seemingly minor effects at the level of a single protein; yet cumulative events across the cell would be profound. For example, breaking the rheostat by knockout of the O-GlcNAc–processing enzymes OGT and OGA is lethal in growing and dividing cells (4, 5, 6, 7). Thus, the O-GlcNAc rheostat is the perfect way for multicellular eukaryotes to link the cell cycle to nutrient availability. Unfortunately, O-GlcNAc does everything, everywhere, all at once; thus, the nature of the O-GlcNAc rheostat makes understanding specific roles for the modification during the cell cycle difficult. The O-GlcNAc rheostat works with other nutrient sensors to prime the cell in Gap phase 1 (G_1_) for growth, modulates the G_1_/S checkpoint, regulates DNA replication in S phase, senses DNA damage in Gap Phase 2 (G_2_), and coordinates division in M phase. Together, the following sections will provide a greater conceptual understanding of how O-GlcNAc influences cell cycle dynamics and cell division providing a broader perspective on how the O-GlcNAc rheostat is a master regulator of growth and division.

## G_1_ phase

The fundamental role of Gap phase 1 (G_1_), the first and longest phase of the cell cycle, is to double cell size and increases organelle copy number. Thus, cells in G_1_ need to capture nutrients shifting the balance between anabolic and catabolic pathways while integrating cellular signaling with metabolism. The binding of growth factors to their cell surface receptors activate mitogenic signaling pathways such as mitogen activated protein kinase pathway inducing the entrance into G_1_ phase and leading to the formation of cyclin D–cyclin-dependent kinase (CDK) 4/6 complexes, an essential kinase complex necessary for progression through G_1_ (8). Counterpoising CDK4/6-cyclin D activity are cyclin complex inhibitors like p21 and p27, which bind to and inhibit CDK4/6-cyclin D (9). Moreover, nutrient-sensing kinases such as mammalian target of rapamycin complex 1 (mTORC1) and AMP-activated protein kinase (AMPK) are crucial mechanisms to integrate and coordinate signaling with nutrient flux (10). During G_1_, two major checkpoints occur: (i) a nutrient-sensing checkpoint involving mTORC1 and (ii) the G_1_/S checkpoint regulated by the retinoblastoma protein (pRb) (11). If environmental conditions are favorable (appropriate levels of growth factors, amino acids, and ATP) during the G_1_, then mTORC1 is activated; the cell synthesizes proteins and lipids gaining size and progresses toward the G_1_/S checkpoint (Fig. 1). Together, these mechanisms dictate the cells’ ability to enter the cell cycle, grow, and divide. Most importantly, all these steps are under the control of the O-GlcNAc rheostat.

## O-GlcNAc dynamics in G_1_

There are two ways to view O-GlcNAc rheostat function through G_1_: 1) at a total cellular O-GlcNAcylation level or 2) at the level of an individual O-GlcNAc site on a given protein. Total cellular O-GlcNAc levels will not always correlate with the G_1_ O-GlcNAcylation status of a single protein. Individual protein substrates can vary dynamically during this phase. When MCF7 breast cancer cells were synchronized into the quiescent G_0_ stage and released into G_1_, global O-GlcNAc levels were low and dramatically increased until S phase entry where total cellular O-GlcNAc levels decreased (12). Using HeLa cells synchronized at the G_1_/S checkpoint, total cellular O-GlcNAcylation decreased slightly into S phase, but extended release of the cells back into G_1_ showed no total cellular O-GlcNAc changes (13). Unfortunately, these apparent G_1_ changes could be an artifact of the synchronization process. Serum starvation is a well-established method for G_0_ synchronization but will also lower most posttranslational modifications. Although G_1_/S synchronization using a double-thymidine block creates a highly synchronous population, release of the block is followed by S phase entry delays while deoxyribonucleotide pools are restored. Changes in nucleotide pools could impact levels of UDP-GlcNAc, the substrate for OGT thereby introducing an additional variable when assessing O-GlcNAc levels during the cell cycle. Despite the evidence suggesting a slight O-GlcNAc decrease at S phase entry, total cellular G_1_ O-GlcNAcylation dynamics are difficult to measure with any certainty.

Although quantitative mass spectrometry studies have not measured specific O-GlcNAc site changes during G_1_, a small cohort of proteins were differentially O-GlcNAcylated through the cell cycle using a combination of mass spectrometry or immunoprecipitation western blots (12). Importantly, many of these O-GlcNAc sites were highly dynamic, but the changes were linked to specific cell cycle phases in a protein-specific manner such as mini-chromosome proteins 3, 6, and 7 (MCM3, MCM6, and MCM7), which organize the replication fork (12). Interestingly, MCM3 and 6 had high O-GlcNAcylation in G_1_ and lower O-GlcNAcylation in S phase, while MCM7 had lower O-GlcNAc in G_1_ and higher O-GlcNAc at S phase (12). This data is exciting because it suggests that OGT and OGA target these mini-chromosome proteins differentially through the cell cycle; however, how and why they target these proteins differentially is a mystery.

Both OGT and OGA inhibitor treatment can influence G_1_ progression. For instance, inhibition of pre-B cells with OGT inhibitor Ac-5SGlcNAc induces cell cycle arrest suggesting the importance of O-GlcNAcylation for G_1_ progression (14); however, Ac-5SGlcNAc is poorly selective for OGT and inhibits other glycosyltransferases (15). Highly selective OGA inhibitors do not appear to slow G_1_ (13), yet OGA inhibition elicits cellular compensation by increasing OGA and lowering OGT expression confounding results (16). Potentially, noncatalytic functions of OGT contribute to G_1_ progression. For example, endogenous OGT with a degron-tag is poorly stable and expressed at low levels, but cells remain viable in a growth-arrested state (17). Replenishing WT OGT restores growth; interestingly, noncatalytic OGT rescued proliferation but at a lower rate when compared with WT OGT (17). Using this model, these data suggest that OGT interactions play essential roles in cell proliferation likely by inducing the formation of multiprotein complexes, altering protein subcellular localization or protein turnover *via* OGT’s scaffolding function.

At least in liver, OGT and OGA were dispensable for regenerative growth. After partial hepatectomy (PHX) in OGT-KO and OGA-KO mice (hepatocyte-specific knockouts), both successfully regenerated a liver; however, liver regeneration in the OGT KO was defective leading to hepatic dysplasia and a neoplastic phenotype (18). After PHX, CyclinD1, CDK4, and phosphorylated pRB increased and sustained high levels up to 28 days. OGA-KO and WT mice decreased the total levels or phosphorylation status of these proteins after 3 days of PHX suggesting sustained cell proliferation only in OGT-KO mice (18). Unique to liver and 3 days after PHX, hepatocyte nuclear factor 4 alpha (HNF4-α), the master transcription factor for hepatocyte differentiation, increased dramatically leading to growth arrest. However, in the OGT KO livers, HNF4-α levels were significantly lower (18). Interestingly, HNF4-α downregulates c-MYC levels, a key transcription factor controlling cell cycle progression (19, 20) suggesting that loss of HNF4-α in the OGT-KO cells will upregulate c-MYC to drive G_1_ progression.

**Figure 2:**
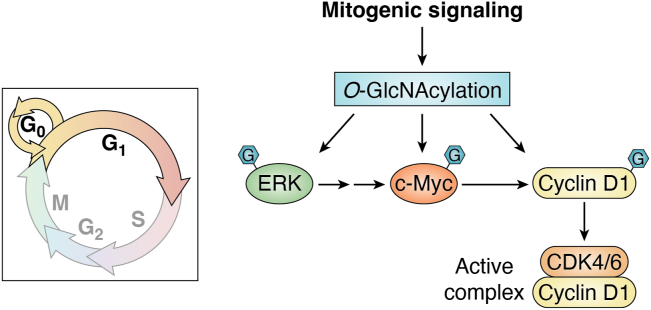
**Entry into G**_**1**_**is facilitated by O-GlcNAc.** Mitogenic signals activate ERK1/2 to phosphorylate the c-MYC transcription factor. Activation of c-MYC induces cyclin D expression. Cyclin D then binds to and activate CKD4/6. O-GlcNAcylation modulates ERK function, c-MYC transcriptional activity, and cyclin D stability. ERK, extracellular activated protein kinase.

**Figure 3:**
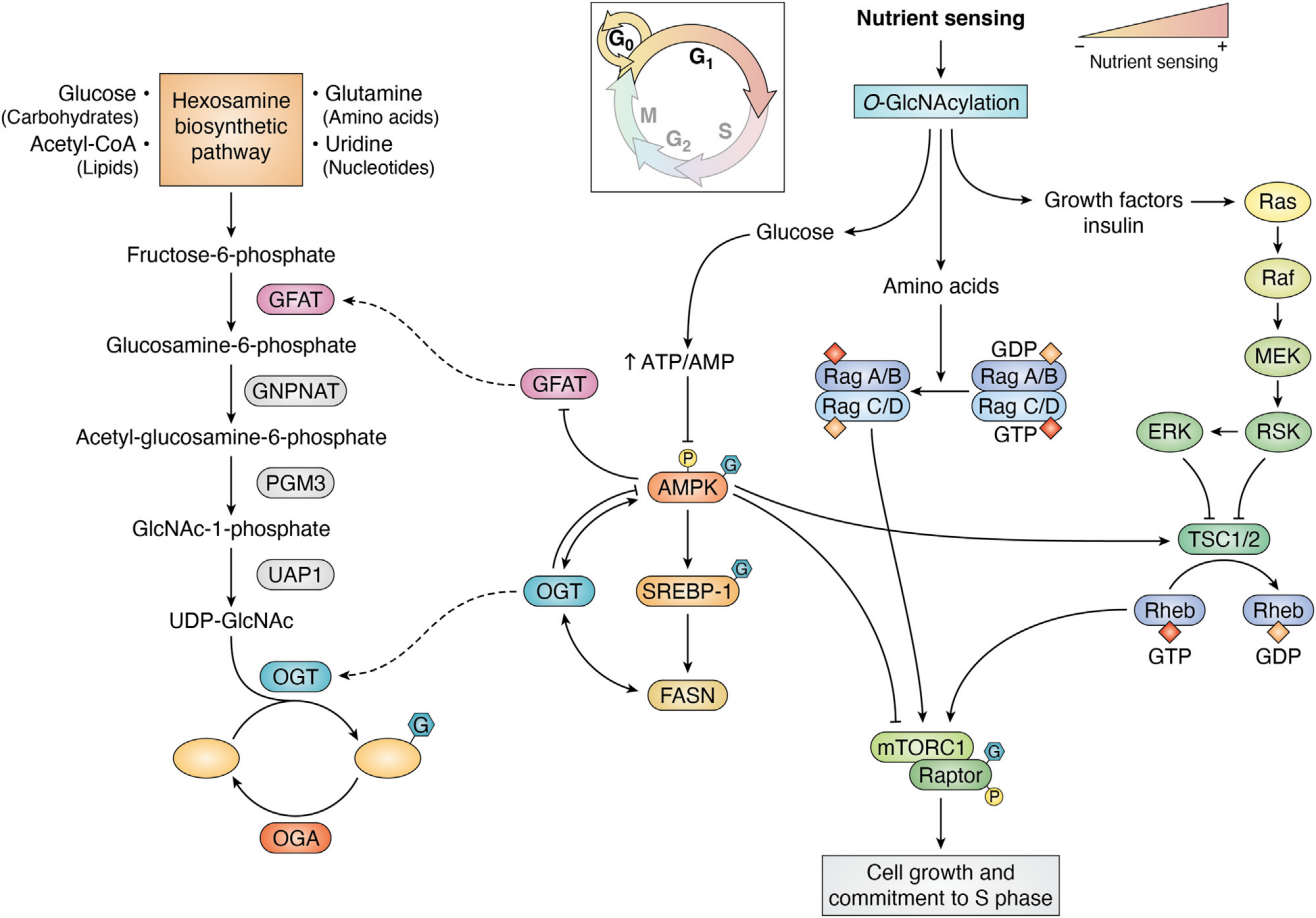
**Interplay between nutrient-sensing pathways drive G1 progression.** UDP-GlcNAc, derived from the hexosamine biosynthetic pathway (HBP), is used by OGT to modify several G_1_ proteins including AMPK. OGT interacts and modifies other nutrient sensors such as the MTOR–AMPK axis and FASN. However, AMPK activation inhibits GFAT, the rate liming enzyme of the HBP. Together, these sensors gauge nutrient availability to induce G_1_ progression. AMPK, AMP-activated protein kinase; FASN, fatty acid synthase; GFAT, glutamine: fructose-6-phosphate amidotransferase; OGT, O-GlcNAc transferase.

The fact that OGA-KO mice lacked the phenotypes observed in OGT-KO mice is probably due to compensatory effects such as degradation of O-GlcNAcylated proteins and lower OGT expression (18). Together, these observations suggest a complex, pleiotropic role for O-GlcNAcylation during the cell cycle and suggests the idea that specific cell types and tissues fine-tune the O-GlcNAc rheostat differently to regulate proliferation. Furthermore, these data suggest that disruption of the rheostat could commit the cell to undergo cycle arrest or neoplastic transformation (Figs. 2 and 3).

## O-GlcNAc control of mitogenic signaling

Upon receiving growth factor (mitogenic) signals from the extracellular environment, cells enter from a quiescent state (G_0_) into G_1_ of the cell cycle (Fig. 1) (21, 22, 23, 24). The signal cascades from the growth factor receptor to the small GTPase Ras and eventually to activation of the map kinase pathway (mitogen activated protein kinase also known as ERK, extracellular activated protein kinase) (25, 26). Downstream phosphorylation targets of ERK, such as the transcription factor c-MYC, modulate the progression of the G_1_ phase (27, 28). ERK increases cell proliferation by inducing c-MYC–mediated expression of the G_1_ cyclin, cyclin D, and represses CDK inhibitor transcription (29). Together, this signaling cascade initiates G_1_ entry and progression. Significantly, the O-GlcNAc rheostat plays critical roles throughout this pathway (Fig. 2).

ERK signaling and O-GlcNAc are intertwined. Alterations to the O-GlcNAc rheostat have a variety of effects on ERK signaling depending on the cell and tissue type. The most consistent impact resulting from O-GlcNAc perturbation is an increase in ERK pathway signaling. Pharmacological increases in O-GlcNAc increase activating ERK phosphorylation in synaptosomes (30), and pathway analysis from RNA-seq data generated in OGA-inhibited neuroblastoma cells predicts increased ERK pathway signaling (31). OGT knockdown lowers phosphorylated ERK in gastric cancers (32), while OGT inhibition depresses ERK1/2 signaling in cardiomyocytes (33). In SH-SY5Y and HeLa cells, OGT and OGA KO or OGA inhibition all amplify ERK signaling (34). In contrast, increased flux through the hexosamine biosynthetic pathway (HBP), which elevates total cellular O-GlcNAc levels, depressed ERK signaling in a cardiac hypertrophic model (35). However, increased flux through the HBP would have a broader range of effects, then targeted O-GlcNAc manipulation (Fig. 3). The upstream ERK kinase MEK2 (36) and ERK1/2 are O-GlcNAcylated (33, 37); although, what effect O-GlcNAcylation has on ERK1/2 and MEK2 activity is unclear. Lastly, increased ERK signaling elevates O-GlcNAcylation, suggesting regulatory cross-talk between O-GlcNAc and ERK signaling (38) (Fig. 2). In most cases, the data suggest that elevated O-GlcNAcylation has a pro-growth affect by increasing ERK signaling. Elevated ERK signaling would then feed into activation of the transcription factor c-MYC.

Several studies have identified an interplay between O-GlcNAc and c-MYC. c-MYC is O-GlcNAcylated at Thr58, a known phosphorylation site (39, 40). When T58 is O-GlcNAcylated, c-MYC ubiquitinylation is prevented, and the half-life of c-MYC is extended (41). Serum starvation increases T58 c-MYC O-GlcNAcylation, but the addition of serum increases T58 phosphorylation (42). Phosphorylated T58 c-MYC is more active; however, the cell quickly degrades c-MYC when phosphorylated (41). Therefore, the cells maintain stable, O-GlcNAcylated, and low-activity c-MYC at G_0_, and upon mitogenic signaling, c-MYC is activated by phosphorylation (Fig. 2). What is unclear is what controls OGA removal of O-GlcNAc on c-MYC to drive c-MYC activation.

Although knockdown or inhibition of OGT led to reduced c-MYC protein levels and failure to initiate growth (43, 44), OGT can affect c-MYC function beyond protein stability. Using ChIP-sequencing analysis, O-GlcNAcylation was elevated at c-MYC promoter regions, suggesting OGT can affect the transcriptional regulation of c-MYC. At the promoter, OGT interacts with the transcriptional co-activator host-cell factor 1 resulting in enhanced host-cell factor 1 interactions at the c-MYC promoter (44). These data demonstrate the transcriptional regulatory function of OGT driving c-MYC target gene expression and enhancing cell growth. Thus, the regulation of c-MYC by O-GlcNAc is multifaceted and combinatory.

Aberrant regulation of c-MYC O-GlcNAcylation contributes to hyperproliferative diseases. c-MYC O-GlcNAcylation in diabetic keratinocytes is increased and promotes hyperproliferation at the margins of diabetic skin wounds (45). Hyperproliferation and c-MYC O-GlcNAcylation was blocked with OGT inhibitor treatment. These data demonstrate that long-term–increased nutrient flux *via* diabetes promotes pro-growth effects of OGT through c-MYC stabilization (45). In prostate cancer, increased HBP flux elevates O-GlcNAc, increasing c-MYC protein stability (43). Additionally, there was a positive correlation between *c-Myc* RNA copy number and OGT expression (43). In this regard, overexpression of c-MYC increases OGT expression, and inhibition of c-MYC decreases OGT expression indicating the integration of nutrient availability and regulation of O-GlcNAc sensing *via* c-MYC activity (46, 47). Additionally, c-MYC regulates the expression of a multitude of metabolic genes upregulating genes involved in glucose and glutamine transport; thus, active c-MYC can drive flux through the HBP to elevate O-GlcNAc (48). Together, these data demonstrate the interconnected relationship between nutrient flux, OGT, and c-MYC and show how proliferative disease can corrupt this relationship with excess HBP flux or mitogenic signaling driving aberrant cell cycle expression.

Although c-MYC regulates a plethora of metabolic and cell cycle genes, c-MYC activation drives the production of the G_1_ cyclin, cyclin D (29). O-GlcNAcylation can influence cyclin D expression in c-MYC–independent ways. Elevated OGT levels promote O-GlcNAcylation of the transcription factor β-catenin promoting cyclin D gene expression (14). Cyclin D itself is O-GlcNAcylated (49). O-GlcNAcylated cyclin D is more stable due to the O-GlcNAc, blocking cyclin D phosphorylation and subsequent ubiquitin-mediated degradation (49). Thus, multiple control points along the ERK–c-MYC–cyclin D axis are under control of the O-GlcNAc rheostat (Fig. 2). However, elevated O-GlcNAc levels almost always promote cell cycle progression; hence, HBP flux is a key driver of this axis and would directly link O-GlcNAc nutrient sensing with other nutrient-sensing pathways at G_1_.

## Nutrient sensing in G_1_: HBP and O-GlcNAcylation

OGT acts as a nutrient sensor dependent upon the availability of its metabolic substrate UDP-GlcNAc. This metabolite is used by many glycosyltransferases (50); however, the high cytoplasmic concentration of the metabolite and OGT’s cellular localization empowers the nutrient-sensing capabilities of O-GlcNAc. The gatekeeper for OGT nutrient sensing is the HBP (50). Four major nutrient pathways converge at the HBP: carbohydrate (glucose), amino acid (glutamine), lipid (acetyl-CoA), and nucleotide (uridine) (Fig. 3). First, the HBP begins with the conversion of fructose-6-phosphate to glucosamine-6-phosphate by the activity of the enzyme glutamine:fructose-6-phosphate amidotransferase (GFAT), the rate-limiting step of this pathway (51, 52). Next, glucosamine-phosphate N-acetyltransferase (GNPNAT) adds an acetyl-group to glucosamine using acetyl-Co-A followed by conversion of GlcNAc-6-phosphate to GlcNAc-1-phosphate by phosphoglucomutase (PGM3) (53). Finally, uridine-N-acetylglucosamine pyrophosphorylase 1 (UAP1) adds a urine to GlcNAc-G-phosphate, generating UDP-GlcNAc (53). Thus, nutrient flux through the HBP controls the nutrient-sensing ability of OGT through G_1_.

The major control point of the HBP is at GFAT because GFAT is modulated by posttranslational modifications and feedback inhibition (54, 55). However, treatment with glucosamine (GlcN) can circumvent GFAT HBP regulation (56). IL-3–dependent hematopoietic cells treated with GlcN in the absence of glucose induced G_1_ by promoting glutamine uptake and metabolism. Despite GlcN treatment inducing an increase in cell size in the absence of glucose, cells failed to proliferate. Moreover, BrdU incorporation showed that cells treated with GlcNAc in the absence of glucose entered S phase yet were unable to complete the cell cycle, indicating HBP activation is sufficient for increasing cell size but insufficient for cell division (57). Unfortunately, GlcN treatment is a double-edged sword because high concentrations can deplete ATP levels blocking growth-promoting affects (56, 58). Interestingly, when human-induced pluripotent stem cell cardiomyocytes were forced into the cell cycle by ectopic expression of cyclinD1, cyclinB1, CDK4 and CDK1, GFAT transcription increased. This led to an elevation in UDP-HexNAc (GlcNAc and GalNAc) levels and activation of the HBP after cell cycle induction (59). Interestingly, OGT overexpression in these cells increased cell cycle entry while OGA overexpression blunted cell cycle entry (59). Together, these data suggest that GFAT activity associated with increased UDP-GlcNAc levels and OGT activation is required for G_1_ progression (Fig. 3).

Alterations to the other HBP enzymes can still affect growth. Gene inactivation by homologous recombination of the enzyme GNPNAT (EMeg32) was embryonically lethal in murine models, and mouse embryonic fibroblasts lacking GNPNAT exhibited defects in cellular proliferation. Adding GlcNAc in the medium or re-expressing GNPNAT restored the UDP-GlcNAc levels and rescued the defect on cell cycle progression (60). Combined, these studies indicate a role for HBP in regulating cell cycle progression and suggest that increased HBP flux regulates OGT’s nutrient sensor potential. Importantly, the HBP flux can synergize with other nutrient-sensing pathways like mTOR and AMPK or signaling pathways like ERK to orchestrate G_1_ progression.

## Interplay between O-GlcNAcylation, mTOR, and AMPK during G_1_ phase

mTORC1 is a central growth hub sensing exogenous growth factors, nutrients, and energy status while controlling cell size and mass by inducing anabolic and inhibiting catabolic processes (Fig. 3). Importantly, mTORC1 is a multiprotein complex activated though growth factor signaling, ATP levels, oxygen levels, and the presence of branched chain amino acids. Nutrient availability induces mTORC1 recruitment to the lysosomal membrane by RagGTPases followed by anchoring *via* the Ragulator complex (61). Once at the lysosome, the lysosomal resident GTPase Rheb activates mTORC1, promoting protein synthesis (61). However, lower branched chain amino acid or growth factor levels suppress mTORC1 activity resulting in G_1_ arrest (62). These metabolic alterations control G_1_ progression *via* the mTORC1-dependent checkpoint responsible for the nutrient sensing required for cell size increases and commitment to S phase (Fig. 3) (63, 64).

Since both O-GlcNAc and mTORC1 are nutrient sensors, then these two pathways can intersect and modulate each other’s function. For example, the mTORC1 pharmacological activator (MHY1485) induced the increase of total cellular O-GlcNAcylation and OGT levels in colon cell lines, while mTORC1 inhibition by rapamycin had the opposite effect, reducing total cellular O-GlcNAcylation and OGT levels in colon cells and various breast cancer cell lines (46, 65). The inhibition of mTORC1 by siRNA silencing of the mTORC1 complex protein Raptor also reduced OGT levels in primary human trophoblast cells (66). Recently, Raptor O-GlcNAcylation enhanced mTORC1 activation by enhancing the interaction of Raptor with Ragulator. This interaction was elevated in high glucose concentrations promoting mTORC1-mediated cell growth and proliferation (67). Thus, these data suggest that mTORC1 activity directly impacts OGT expression, O-GlcNAc regulates mTORC1, and both mTORC1 and OGT are positive regulators of cell growth with nutrient excess–increasing activity of both sensors.

Since increased OGT activity is linked to nutrient excess, we would expect high O-GlcNAc levels or OGT overexpression to coincide with mTORC1 activity. In fact, increased O-GlcNAcylation either by pharmacologic inhibitor of OGA (Thiamet-G, TMG) or OGT overexpression induced inactivation of mTORC1 regulator AMPK (as measured by reduced Thr172 phosphorylation on AMPK). Subsequently, mTORC1 activation was increased (as measured by higher Ser2448-mTOR phosphorylation and phosphorylation of mTORC1 substrate Ser424 of S6 Kinase) in LoVo colon cancer cell line (68). Again, these data suggest that elevated total cellular O-GlcNAcylation is a positive regulator of mTORC1.

Of course, nothing with O-GlcNAc is ever straightforward. In an elegant genome-wide CRISPR-Cas9 study from mouse embryonic stem cells containing an inducible OGT knockout (mESC, *Ogt iKO*), 115 CRISPR targets restored G_1_ phase progression and cell growth (69). Most targets were related to mitochondria function, respiratory electron transport, or mTORC1 signaling (69). OGT deficiency increased proteasome activity resulting in a global increase in amino acid levels (including the mTORC1 regulators leucine, isoleucine, and valine), promoting translocation of mTORC1 to the lysosome and leading to increased phosphorylation of S6 kinase targets serine 235/236 of S6 ribosomal protein (69). While proteasome inhibition reduced mTORC1 lysosomal translocation and activity, inhibition of mTORC1 or targeted knockdown of mTORC1-associated proteins restored G_1_ progression and growth (69). Importantly, increased amino acid levels and subsequent mTORC1 activation also occurred in *Ogt iKO* CD8^+^T cells, suggesting that this effect is not cell type–specific. Together, these results clearly indicate a mechanism where in the absence of OGT, the proteasome becomes highly active, generating a significant increase in intracellular amino acid levels resulting in mTORC1 activation, which disturbs cell cycle progression and decreases cellular proliferation and viability (69). Thus, low O-GlcNAc levels correlating with low nutrient flux can still activate mTORC1 by artificially elevating amino acid levels though protein degradation mimicking nutrient excess.

Fatty acid synthesis is essential for doubling membrane volume in G_1_, and fatty acid synthase (FASN) is linked to both O-GlcNAcylation and mTORC1 activity (70). In liver HepG2 cells during G_1_, both O-GlcNAcylation and FASN levels increased as well as an increase in OGT interactions with FASN. OGT interactions with FASN were reduced during S and M phase. Inhibition of OGT or OGT knockdown reduced FASN expression (70). Thus, OGT activity and interaction with FASN promotes G_1_ progression. Furthermore, mTORC1 activity is required for increased FASN expression at G_1_. FASN levels increase in accordance with the rise of mTORC1 activity in G_1_, and mTORC1 inhibition reduces FASN expression. Finally, the treatment with FASN inhibitor (C75) reduced O-GlcNAcylation and OGT levels, as well as inactivated mTORC1 (70). These data demonstrate an important interplay between mTORC1, OGT, and FASN in which each nutrient-sensing complex interacts synergistically with each other and activation or inhibition of mTORC1 induces increased or decreased O-GlcNAcylation, respectively. These data also argue that nutrient sensors are connected and coordinate cellular response to an extracellular nutrient flux (Fig. 3).

AMPK, a crucial nutrient-deficit sensing enzyme complex, regulates cell metabolism in opposition to mTORC1 (Fig. 3) (71). The activation of AMPK induces catabolic and inhibits anabolic processes. AMPK is a heterotrimeric complex composed of a catalytic α-subunit and two regulatory subunits, β and γ, where the γ-subunit binds to AMP, ADP, and ATP (71). AMPK activation occurs by binding to AMP and ADP, while inhibition occurs by binding ATP; thus, the AMPK is responsive to intracellular energy levels by sensing the ratios of AMP:ATP and ADP:ATP (71).

Besides opposing mTORC1 function, there is extensive cross talk between AMPK and O-GlcNAcylation (72). In rat C2C12 muscle myotubes, AMPK phosphorylates OGT at Thr-444, inducing nuclear localization and altering substrate selectivity of OGT. Furthermore, both the α- and γ-subunits of AMPK are dynamically O-GlcNAcylated (72). AMPK activation, as measured by phosphorylation of T172 of AMPK (a critical activation site), is induced either by AICAr treatment (AMP mimetic used as AMPK activator) or by glucose deprivation, which increases the O-GlcNAcylation of γ-subunit suggesting that AMPK O-GlcNAcylation is associated with increased AMPK activity. However, acute treatment with an OGA inhibitor, which increases total cellular O-GlcNAcylation, blunts the activation of AMPK either by AICAr or glucose deprivation, suggesting the cycling of O-GlcNAc on AMPK is more important for activation than a static modification (72). Together, these results demonstrate a nutrient sensor axis between AMPK, mTOR, and OGT regulating metabolic function.

Moreover, FASN gene expression is linked to AMPK *via* O-GlcNAcylation of the transcription factor sterol regulatory element binding protein 1 (SREBP-1) in an AMPK-dependent manner (73). First, OGT silencing or inhibition decreased SREBP-1 expression and target gene transcription including FASN (73). Loss or reduced SREBP-1 function results in G_1_/S arrest, corroborating the need for fatty acid biosynthesis in G_1_ (74). Second, SREBP-1 O-GlcNAcylation is dependent on AMPK activation because the reduction of SREBP-1 observed by silencing OGT was not observed in AMPK null murine embryonic fibroblasts (73). Thus, AMPK and OGT synergize to modulate FASN expression through SREBP-1, suggesting a complex interplay between FASN, mTORC1, OGT, and AMPK to sense nutrients in G_1_ (Fig. 3).

Interestingly, AMPK also works upstream of OGT by phosphorylating GFAT at Ser243, reducing GFAT activity (75). In fact, AMPK inhibition of GFAT is critical to block cardiac hypertrophy. The induction of cardiomyocyte hypertrophy by phenylephrine was accompanied by increased O-GlcNAcylation (76). AMPK mechanistically reduces total cellular O-GlcNAcylation by phosphorylation of GFAT at Ser243 followed by reduced HBP flux (76). The AMPK activators AICAr and A769662 reversed the hypertrophy induced by phenylephrine, but inhibition of OGA (PUGNAc, an OGA inhibitor) counteracts the effect of AMPK activators showing the importance of elevated O-GlcNAcylation for hypertrophy. Pharmacological activation of AMPK *via* A769662 induced the reduction of total cellular O-GlcNAc, while the AMPKα2 KO mice hearts display increased total cellular O-GlcNAcylation indicating that AMPK and O-GlcNAcylation levels oppose each other *in vitro* and *in vivo*.

Clearly, nutrient sensors are essential for G_1_ progression, and nutrient sensors act synergistically to control growth (Fig. 3). While AMPK and mTORC1 act as off and on switches, respectively, O-GlcNAcylation acts as a nutrient rheostat in G_1_ fine-tuning each pathway for optimal growth outcomes. This additional complexity will require new and sophisticated experimental approaches to tease apart the interplay between these nutrient sensors. Likely, unbiased multi-Omic studies will uncover a deeper mechanistic understanding of the rich interplay between nutrients and nutrient sensors in controlling growth.

## O-GlcNAcylation regulates the G_1_/S transition

**Figure 4:**
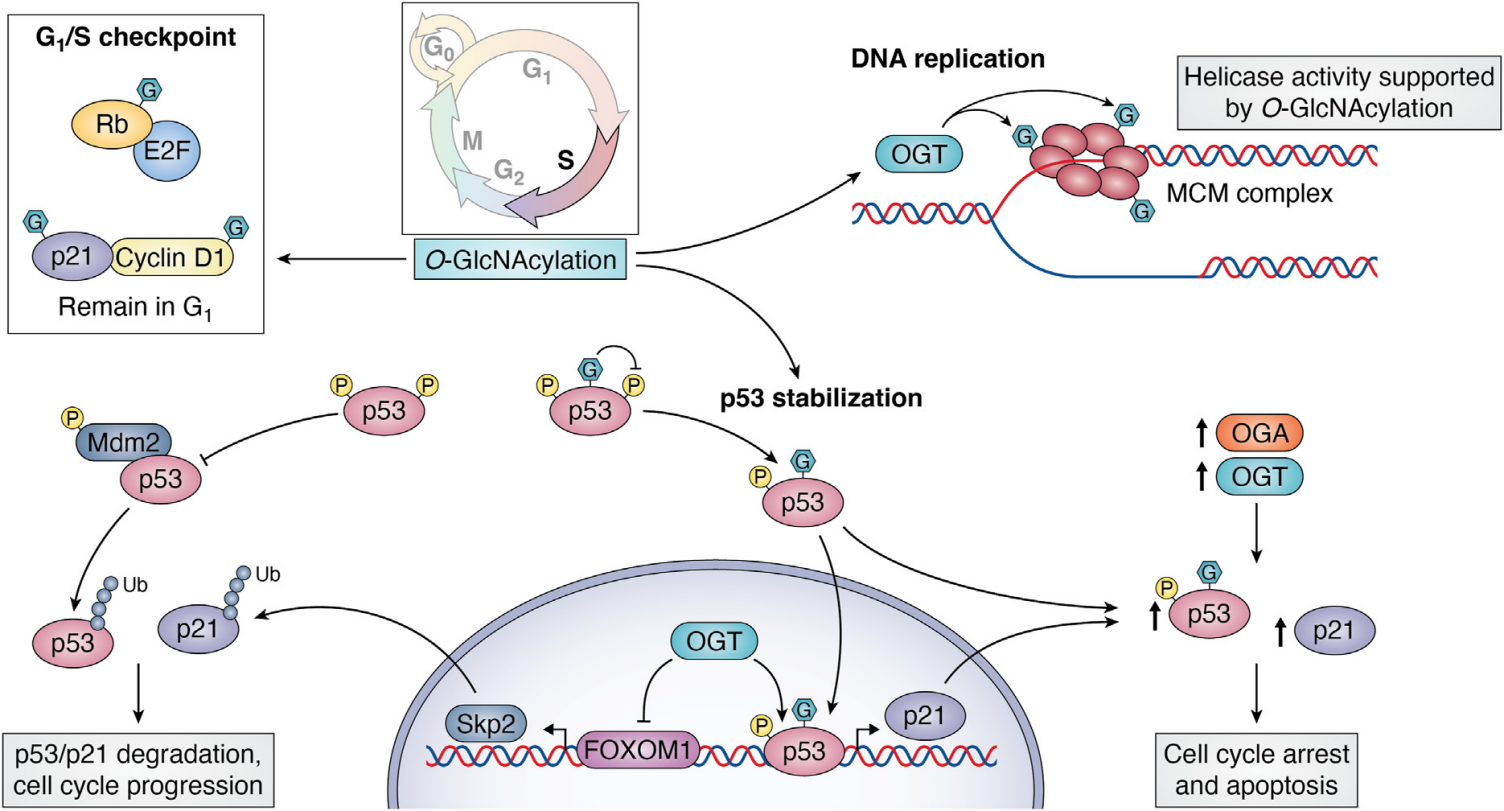
**O-GlcNAc stabilizes DNA replication machinery and modifies p53 stabilization**. At the G_1_/S checkpoint, pRB is O-GlcNAcylated and contributes to checkpoint regulation. S phase entry is characterized by DNA synthesis and replication in preparation for mitosis. O-GlcNAc enhances the ability of the mini-chromosome complex (MCM) to bind chromatin and induce helicase activity, thereby permitting DNA replication. Errors in replication are sensed by p53, a key checkpoint sensor in G_2_. p53 is degraded when bound to MDM2. Phosphorylation of p53 prevents MDM2 association and degradation; O-GlcNAc inhibits phosphorylation, increasing the stability of p53. Once stable, p53 induces transcription of p21. p21 is an inhibitor of CDKs and prevents cell cycle progression. OGT or OGA overexpression can directly induce p21 expression and increase p53 stabilization. OGT, O-GlcNAc transferase; OGA, O-GlcNAcase; pRb, retinoblastoma protein.

A major checkpoint on cell growth is at the G_1_/S transition. The pRB and related family members (p107, RBL1 and p130, RBL2) are transcriptional repressors active in their hypo-phosphorylated state that bind to transcription factors (principally the E2F family) at cis-regulatory elements within the genome blocking expression of genes involved in S phase progression (77, 78, 79). Canonically, the checkpoint is released when pRB becomes hyper-phosphorylated by CDK4/6-cyclin D and can no longer bind E2F proteins (80, 81, 82). Freed E2F transcription factors then induce S phase gene expression (80). Importantly, pRB is O-GlcNAcylated. pRB O-GlcNAcylation blocks phosphorylation of pRB, promoting pRB binding with E2F, which results in cell cycle arrest at the G_1/_S checkpoint (83) (Fig. 4). An interesting knowledge gap is the relationship of O-GlcNAcylated pRB levels with p21 levels. p21 is a CDK inhibitor that binds to cyclin D1–CDK4 and S phase cyclin E–CDK2 complexes preventing G_1_ progression (84). p21 can be stabilized with elevated O-GlcNAcylation independent of p53 (85). Thus, more p21 bound to cyclin D due to higher O-GlcNAc levels would prevent pRB phosphorylation and the release of E2F supporting cell cycle arrest. As an effective rheostat of nutrient availability, O-GlcNAcylation can influence the G_1_/S transition by affecting pRB phosphorylation or indirectly by influencing the levels of either cyclin D or p21.

## S phase

S phase is reserved for the synthesis of new DNA (Fig. 1). As with the other phases of the cell cycle, how cellular O-GlcNAcylation levels change during this phase is unclear. Elevated O-GlcNAc *via* increased flux through the HBP, glutamine availability, or OGA inhibition leads to prolonged S phase and depression of cell division (12, 13), while loss of OGA expression also prolonged S phase and slowed progression to M phase (86). Overall, the data suggest that higher total cellular O-GlcNAc levels slow S phase progression. However, direct influence of O-GlcNAc on S phase occurs through multiple mechanisms including regulating DNA replication, which complicates how changes in total cellular O-GlcNAc levels impact progression.

## O-GlcNAc regulates the replication fork

DNA replication is a complex coordination of histone modifications, opening of the DNA, assembly of the replication complex at the replication origin site, and continuous synthesis of daughter stranded DNA (87). This process is regulated by protein synthesis, posttranslational modifications, and protein degradation (87, 88). Key to controlling replication is the mini-chromosome complex (MCM). The MCM is composed of several subunits creating a ring-shaped structure with ATPase-associated activity that varies depending on the subunits involved including subunits having DNA helicase activity (89, 90, 91, 92, 93, 94, 95, 96). Not unexpectedly, several MCM proteins including MCM 2/3/5/6/7 are O-GlcNAcylated proteins (12, 37, 97). Interestingly, these proteins are tightly regulated by their phosphorylation status, promoting loading of the complex onto chromatin (88, 92). The O-GlcNAcylated MCM preferentially binds to chromatin, and OGT strongly associates with the chromosome-bound portion (97). OGT associated with MCM proteins 2/3/5/6/7 with the highest affinity for MCM3, 6 and 7. Reducing OGT expression results in less efficient binding of MCM7 to chromatin and decreased stability of the MCM complex, suggesting OGT activity is essential to recruiting and ultimately permitting MCM ATPase activity and therefore, DNA replication during S phase (97). However, disruption of O-GlcNAc homeostasis does not completely block MCM activity and only modestly slows S phase progression resulting in decreased cell growth (Fig. 4) (97). What is unclear is how the dynamic interplay between O-GlcNAcylation and phosphorylation on the MCM impacts replication fork function. Recent advances in O-GlcNAc mass spectrometry site mapping (98) could lead to sophisticated mutagenesis studies to tease this interplay apart leading to a greater understanding of the mechanistic switching between these two modifications.

## The O-GlcNAc rheostat drives alternate fates of p53 signaling from G_1_ to G_2_

A critical regulator for the progression of the cell cycle from G_1_ through S phase and into G_2_ is the transcription factor p53. A high-fidelity S phase with DNA free of mutations is essential for cell survival and growth. Errors in DNA replication initiate protein cascades to repair DNA mutations or breaks and to slow or stop cell cycle progression (99). As part of this cascade, DNA damage–sensing kinases activate p53 to promote the expression of genes that block cell cycle progression such as p21 (100). While p53 exerts dominant checkpoint effects at G_1_, G_2_, and M phase, it is predominantly present at the beginning of G_2_ (101). Once DNA replication is complete, DNA integrity is checked, and if errors have occurred, kinases such as ATM and ATR phosphorylate p53-binding protein and ubiquitin ligase complex protein MDM2 and p53 to prevent their association (102, 103, 104, 105). Phosphorylation of p53 renders it “stable” and it translocates to the nucleus binding to DNA and inducing expression of several downstream targets (106). Expression of p53 induces genes facilitating DNA repair allowing the cell to mend any replication errors and proceed through the cell cycle (107). If repairs are not made, p53 induces expression of apoptosis gene programs (107). Not unexpectedly, p53 is O-GlcNAcylated (108) (Fig. 4).

Serine 149 on p53 is O-GlcNAcylated, and the O-GlcNAcylated form of p53 has lower phosphorylation at T155 preventing MDM2 associating with p53 leading to p53 stabilization (109). Even though O-GlcNAcylation at S149 is not reciprocal to phosphorylation at T155, O-GlcNAc is a large enough modification to sterically block phosphorylation at T155. Stabilization and nuclear translocation of p53 results in prolonged cell cycle arrest after doxorubicin treatment and eventually apoptosis (109).

One weakness of our understanding of site-specific p53 O-GlcNAcylation is the inability to detect GlcNAc on WT p53 without inducing an increase in O-GlcNAc or introducing DNA-damaging agents. However, this informs the field on p53 stabilization and its consequences in diseased states with elevated O-GlcNAc such as hyperglycemia. O-GlcNAc–mediated stabilization of p53 in hyperglycemia contributes to diabetic retinopathy *via* increased apoptosis in retinal pericytes (110). Induction of apoptosis in hyperglycemic mice led to coronary endothelial cell death *via* O-GlcNAcylated p53 stabilization (111). Subsequently, microvascular disease developed from increased cardiac load and damage reversible by decreasing O-GlcNAc with overexpression of OGA resulting in less p53 stabilization (111). Thus, not only is p53 modified by O-GlcNAc but it profoundly tips the scale between health and disease across various tissues.

Consequently, nutrient flux through the HBP affects p53 stability by either inducing apoptosis or by activating downstream expression of p53 target genes like the CDK inhibitor p21 (Fig. 4). p21 is directly increased in response to disruption of the O-GlcNAc rheostat. In synchronized HeLa cells, OGT and OGA overexpression had lower levels of p21 as cells moved though the cell cycle (13); but in unsynchronized ovarian and neuroblastoma cancer cells, OGT and OGA overexpression elevated p53 levels as well as p53 target genes MDM2 and p21 (112). OGA knockdown in ovarian cells lowered MDM2 protein levels and increased p21 proteins levels, while OGA inhibition also increased p21 protein levels. Likely, O-GlcNAc influences p53 levels and function at multiple checkpoints. Not only can O-GlcNAc stabilize p53 but changes in O-GlcNAc affect MDM2 phosphorylation and p53 acetylation (K382). MDM2 phosphorylation reduces p53 binding and both OGT and OGA overexpression increases MDM2 phosphorylation (112). Both OGT overexpression and OGA knockdown increase p53 acetylation, promoting target gene expression (112). Interestingly, when OGT is inhibited, p21 levels increase in a p53-independent manner, decreasing cell proliferation without increasing cell death (85). OGT inhibition or OGT knockout lowers the expression of the transcription factor Forkhead Box M1 (FOXM1) (85). FOXM1 regulates cell cycle progression by transcribing genes that compose the SCF (Skp2-Cks1) ubiquitin ligase complex (113). OGT inhibition or OGT knockdown lower SKP2 protein levels preventing p21 ubiquitin ligase–mediated proteasome degradation (85). The regulation of p21 is another example of the pleotropic nature of the O-GlcNAc rheostat in which O-GlcNAc regulates gene expression through p53 or p21 protein stability *via* ubiquitin ligation. Thus, these experiments argue for greater nuance in interpreting data after modulation of the rheostat and suggest experiments using sophisticated multi-omic methods combined with novel bioinformatics to unify cellular changes caused by O-GlcNAc rheostat manipulation.

## M phase

Mitosis (M phase) is the last stage in the cell cycle and is characterized by the formation of the mitotic spindle, which pulls the replicated chromosomes apart leading to cell division (114, 115). The four stages of the M phase are the following: prophase, metaphase, anaphase, and telophase. Prophase is characterized by increased CDK1 activity after binding to cyclin B, nuclear envelope breakdown, chromosome condensation, and the construction of a complex tubulin structure in which α- and β-tubulin start to elongate toward the condensed chromosomal DNA from a γ-tubulin base known as the centriole (114, 115). In metaphase, the mitotic spindle is complete with tubulin fibers connecting the centrosome (the centrioles and the surrounding proteinaceous area) to the chromosomal DNA at centromere histones *via* a multiprotein complex known as the kinetochore (114, 115). Anaphase sees the condensed chromosomes pulled apart and lower CDK1 activity and cyclin B degradation. Finally, telophase marks the reformation of the nuclear envelope with actin and escort proteins pinching the daughter cells apart at the midbody (the area connecting the two daughter cells) in a process called cytokinesis (114, 115). Furthermore, M phase is heavily regulated by several M phase kinases, M phase–specific ubiquitin ligase complexes, and by the spindle assembly checkpoint (114, 115) (Fig. 1). Not surprisingly, the O-GlcNAc rheostat fine-tunes every step of M phase (13, 116, 117).

## O-GlcNAc dynamics in M phase

As with the other phases of the cell cycle, the total cellular O-GlcNAcylation level as cells move through M phase is unclear. Synchronization into prophase using microtubule polymerization blockers like nocodazole or G_1_/S release from thymidine have produced confusing results with O-GlcNAc being high in some experiments and low in other experiments regardless of synchronization method or O-GlcNAc detection method (13, 118, 119, 120). M phase O-GlcNAcylation changes are far less robust than the M phase phosphorylation increase from activation of CDK1/cyclin B and other mitotic kinases (121, 122, 123). Nonetheless, synchronization artifacts affecting both phosphorylation and O-GlcNAcylation are difficult to control. On the other hand, without synchronization in tissue culture cells, interphase (G_1_, S, and G_2_) would dominate the cell population making M phase phosphorylation or O-GlcNAcylation dynamics impossible to measure.

Meiotic prophase in oocytes does hint at lower O-GlcNAc levels. Early studies in *Xenopus* oocytes demonstrate a decrease in O-GlcNAc concomitant with an increase in OGA activity as oocytes mature and arrest at stage IV oocytes (germinal vesicle) in meiotic prophase 1 (124, 125). Additionally, blocking O-GlcNAc turnover in *Xenopus* oocytes by microinjecting galactosyltransferase to cap O-GlcNAc with galactose leads to cell death after stimulation with progesterone that drive oocytes to mature into meiotic metaphase 2 (germinal vesicle breakdown, GVBD) (126). Moreover, injecting free GlcN or incubating cells in GlcN or the OGA inhibitor PUGNAc slow *Xenopus* oocyte maturation into meiotic metaphase 2 (124, 127, 128), although, progesterone stimulation increases O-GlcNAc levels from GV to GVBD (37, 125, 127). Moreover, OGT microinjection in stage VI oocytes increases progesterone-mediated GVBD and inhibition of OGT blocks maturation (129, 130). Certainly, OGT is critical for M phase progression and localizes to both the mitotic and meiotic spindle (13, 37, 131); interestingly, OGA localizes to the cortex of meiotic cells and OGA inhibition has only a mild reductive effect on GVBD formation (124, 131, 132). However, OGA inhibition does robustly interferes with fertilization leading to polyspermy and improper sperm head decondensation (132). These data demonstrate a cellular need to dynamically cycle O-GlcNAc on and off proteins as M phase progresses and interference with the O-GlcNAc rheostat leads to impaired M phase exit in both mitotic and meiotic cells.

**Figure 5:**
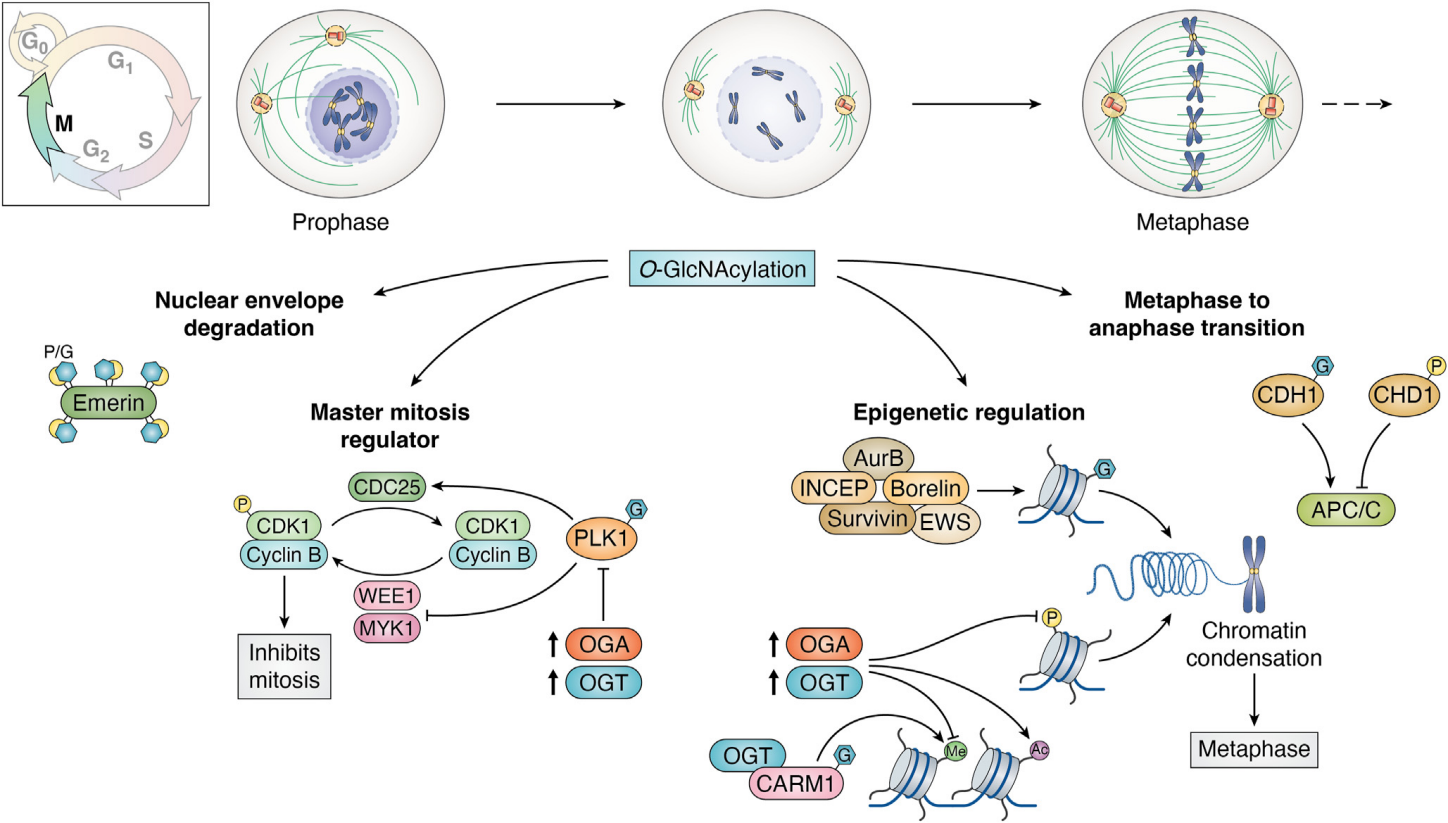
**O-GlcNAc influences mitosis *via* O-GlcNAcylation of critical mitotic mediators**. At prophase, overexpression of OGT or OGA modifies CDK1-cyclin B activity *via* inhibition of PLK1, ultimately inhibiting mitotic entry. Emerin is reciprocally O-GlcNAcylated and phosphorylated, and Emerin O-GlcNAcylation affects degradation of the nuclear envelope during prophase. The metaphase to anaphase transition is activated by APC/C, and O-GlcNAcylation of CDH1 is required to activate this transition. Chromatin condensation during metaphase is regulated in part by O-GlcNAcylation of histones or O-GlcNAc modulation of other epigenetic marks. Overexpression of OGT or OGA increases chromatin acetylation and decreases methylation. OGT, O-GlcNAc transferase; OGA, O-GlcNAcase.

If we lived in a far less complex world, phosphorylation would go up in M phase and O-GlcNAcylation would decrease; however, this system-wide yin-yang principle does not hold up under scrutiny (133). For example, when M phase phosphorylation was quantified after OGT overexpression, 7% of the phosphorylation sites increased while 17% decreased; of the phosphorylation sites that decreased, most were linked to changes in CDK1 activity (117). Looking at an individual protein’s O-GlcNAcylation or phosphorylation might be a better indicator of a functional change or competition between posttranslational modifications. For example, Emerin, a nuclear envelope protein component of the lamina network, is both extensively phosphorylated and O-GlcNAcylated (134). Five O-GlcNAc sites on Emerin are also mitotic phosphorylation sites linked to nuclear envelope breakdown during prophase; thus, the interplay of phosphate and O-GlcNAc at these sites during mitosis likely fine-tunes the function of Emerin as the envelope disassembles (Fig. 5) (134). Another example of M phase–specific O-GlcNAcylation is on the protein CDH1 (Cdc20 homologue 1), which activates the anaphase promoting complex (APC/C) (135). The APC/C is a ubiquitin ligase complex essential for the degradation of multiple mitotic substrates driving the transition from metaphase to anaphase (136). Phosphorylation of CDH1 blocks binding and APC/C activation while O-GlcNAcylation increases CDH1 APC/C binding and activation (135). At the protein level, the dynamics of phosphorylation and O-GlcNAcylation are critical to regulate a specific protein. Likely, these two post-translational modifications (PTMs) can either antagonize or compliment a protein’s function. In this regard during M phase, kinases, phosphatases, OGT, and OGA harmonize to modulate M phase dynamics.

Pharmacologic or genetic alterations to O-GlcNAc greatly impact the mechanics of M phase progression. Overexpression or loss of OGT or OGA cause errors in the assembly of the mitotic spindle, delay M phase progression, and disrupt cytokinesis (13, 18, 86, 116, 137). Cytokinesis errors include lagging chromosomes and catastrophic midbody collapse leading to binucleated and aneuploid cells. In normal cells, loss of OGT or OGA is lethal (6, 17); however, cancer cells which tend to have reduced mitotic checkpoint function and impaired p53 response survive with increased aneuploidy. Moreover, many cancers have elevations in OGT expression, and one potential benefit would be to increase aneuploidy (138, 139, 140, 141). Although aneuploidy can be catastrophic for cell survival, the increased and rearranged DNA content in these cells can provide a growth advantage to the cancer cell (142). In fact, most cancer cells show high levels of aneuploidy (142). How changes in the O-GlcNAc rheostat promote aneuploidy can be understood by how O-GlcNAcylation affects mitotic kinase function, spindle formation, mitotic epigenetic marks, and daughter cell segregation.

## The O-GlcNAc rheostat control mitotic kinases function

Although several kinases are essential for M phase, the master driver of M phase progression through prophase and metaphase is CDK1 bound to Cyclin B (143, 144). Binding of CDK1 to Cyclin B is essential for CDK1 function; however, cells exert regulatory control on CDK1 through inhibitory phosphorylations at threonine-14 and tyrosine-15 *via* the kinases WEE1 and MYT1 (145, 146, 147, 148, 149). The phosphatase CDC25 removes these inhibitory phosphorylations from CDK1 thus forming a kinase/phosphatase switch controlling CDK1 activity (149, 150). Interestingly, this switch is under control of the O-GlcNAc rheostat. Overexpression of OGT, OGA, or OGA knockdown led to an increase in CKD1 tyrosine inhibitory phosphorylation (86, 117, 137) (Fig. 5). With OGT overexpression, MYT1 protein levels are higher, and Cdc25 mRNA levels are lower. These data directly link CDK1 activity and M phase initiation with the O-GlcNAc rheostat placing mitosis directly under control of O-GlcNAcylation.

Moreover, this CDK1 switch is controlled by the mitotic kinase polo-like kinase 1(PLK1), which phosphorylates and inhibits WEE1 and MYT1 while simultaneously activing CDC25 (151). PLK1 protein, mRNA, and activating phosphorylation on PLK threonine 210 levels are lower with OGT overexpression (117). Additionally, OGA overexpression also lowers PLK1 protein levels (137). PLK1 interacts with OGT and is O-GlcNAcylated at threonine 291 (117, 152) (Fig. 5). Interestingly, O-GlcNAc T291A PLK1 mutant increased cell growth, suggesting the O-GlcNAc site on PLK1 decreases activity (152). Conversely, treatment with OGA inhibitor TMG did not significantly affect CDK1 Y15 phosphorylation or PLK1 levels, suggesting that higher total cellular O-GlcNAcylation levels is not what drives O-GlcNAc regulation of CDK1. TMG rescues the decrease in PLK1 levels when either OGA or OGA are overexpressed suggesting the cycling of O-GlcNAc controls PLK1 levels. Unfortunately, the pleotropic nature of O-GlcNAc manipulation experimentally makes this a difficult question to answer.

**Figure 6:**
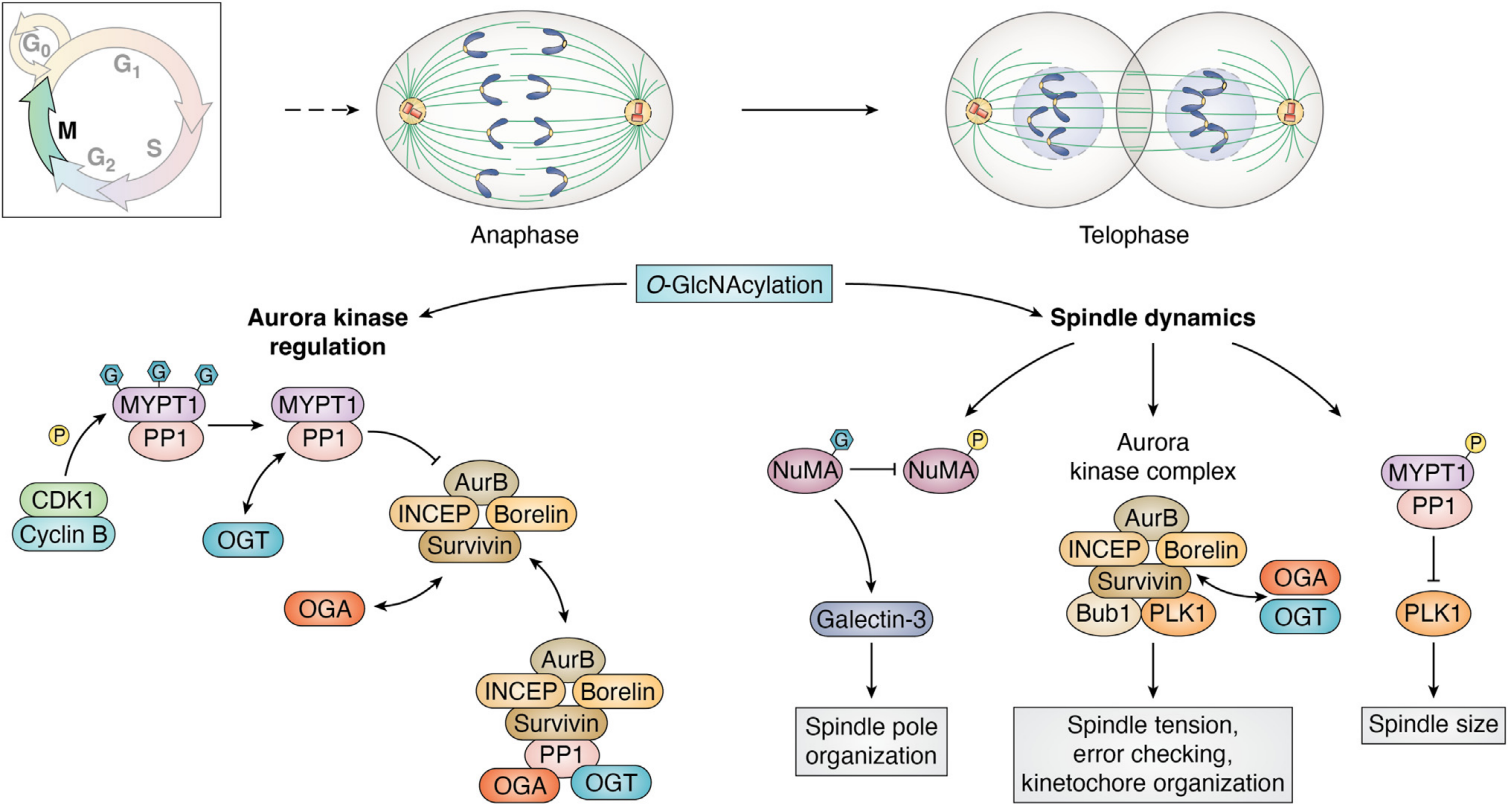
**O-GlcNAcylation controls spindle assembly/disassembly.** Spindle dynamics are tightly regulated by AurB which interacts with OGT and OGA. AurB localizes at the spindle and is regulated by MYPT1 and PP1; however, MYPT1 O-GlcNAcylation alters this inhibition. Furthermore, EWS associates with AurB at the spindle, and loss of OGA affects this interaction. O-GlcNAcylated NUMA inhibits NUMA phosphorylation, disturbing the organization of the mitotic poles. Several spindle and mitotic regulatory proteins are O-GlcNAcylated, and disruptions in the O-GlcNAc rheostat result in aneuploidy. EWS, Ewing sarcoma; OGT, O-GlcNAc transferase; OGA, O-GlcNAcase; PP1, protein phosphatase 1.

The Aurora family of mitotic kinases (AurA, AurB, and AurC) are kinases that drive spindle formation, midbody formation, and cell division (153, 154, 155), and the interplay between the O-GlcNAc rheostat and the aurora kinases control these processes (116, 137). AurB is located at the spindle midzone and then migrates to the cleavage furrow at the midbody to organize cytokinesis (155). AurB interacts with INCENP (inner centromere protein), survivin, and borelin to form the chromosomal passenger protein complex (156). AurB function is antagonized by protein phosphatase 1 (PP1) (157); importantly, OGT and OGA interact with AurB and PP1 at the cleavage furrow (116) (Fig. 6). Together, this signaling complex modulates the PTM status of mitotic substrates; for example, the O-GlcNAcylation and phosphorylation status of the intermediate filament protein vimentin is regulated by this complex (116). Dual regulation of a protein by phosphorylation and O-GlcNAcylation appears common at M phase. OGT interacts with mitotic kinase checkpoint kinase 1, and together, the enzymes modulate intermediate filament modifications with OGT to control cytokinesis (158).

Genetic or pharmacologic manipulation of O-GlcNAc did not affect AurB localization or function at the midbody (86, 116); however, AurB spindle localization is impaired (86, 137). AurB interacts with the Ewing sarcoma protein to facilitate spindle localization (159, 160), but the overexpression of OGT and OGA or OGA being knocked down alters EWS and AurB spindle localization (86, 137); in turn, this can lead to aneuploidy (86, 161). Alternatively, pharmacological inhibition of AurB causes the loss of OGT to the midbody. Although OGT is not a known substrate for AurB and AurB is not a known substrate for OGT, these proteins can modulate each other’s localization to control spindle and midbody function. Together, these data suggest a super-molecular complex composed of OGT, OGA, mitotic kinases, phosphatases, and numerous accessory proteins to regulate the M phase posttranslational environment while disruptions to O-GlcNAcylation and phosphorylation environment lead to aneuploidy and other mitotic defects (Fig. 6).

## The O-GlcNAc rheostat modulates mitotic epigenetics

A key substrate of the AurB/PP1 complex at the spindle midzone is histone H3 serine 10 (H3S10) (162, 163, 164). Phosphorylation of this site drives chromosome condensation at metaphase (163, 164). However, disruptions to the O-GlcNAc rheostat impact H3S10-phosporylation. OGT and OGA overexpression and OGA knockdown all lower H3S10 phosphorylation (86, 137, 165, 166). What is unclear is how the O-GlcNAc rheostat modulates H3S10’s modification state. Are changes in OGT and OGA function impacting kinase activity or is histone O-GlcNAcylation directly blocking phosphorylation? Histones are O-GlcNAcylated including H3 (165, 167), but no O-GlcNAc sites have been definitively mapped to H3S10. Moreover, histone O-GlcNAcylation increases at M phase and appear highest in prophase and metaphase (166, 167, 168). Clearly, future experiments such as *in vitro* AurB activity assays on O-GlcNAcylated histone substrates are needed to answer this question.

Moreover, other mitotic histone modifications are disrupted by OGT overexpression. OGT overexpression decreases mitotic H3K9-2 methylation, increases H3K9 acetylation, lowers H3R17-2 methylation, and increases H3K27-3 methylation. Importantly, OGT associates with both myosin phosphatase targeting subunit 1 (MYPT1), a PP1 targeting protein, and coactivator-associated arginine methyltransferase (CARM1) and is targeted to substrates by these proteins (169). Hence, OGT can be targeted to mitotic epigenetic substrates by MYPT1 and CARM1. Furthermore, CARM1 is O-GlcNAcylated, methylates H3R17 (169, 170, 171), and is found at the centrosomes during metaphase (166). Thus, the mitotic histone code is under the influence of the O-GlcNAc rheostat (Fig. 5). Disruptions to the O-GlcNAc rheostat tilt the balance of chromatin modifications at the spindle toward spindly assembly errors, mitotic arrest, or aneuploidy.

## Spindle dynamics are regulated by O-GlcNAcylation

The mitotic spindle is a wonder of biological architecture. Formed by interlocking cables of α and β-tubulin growing from a γ-tubulin core in the centrosome, the cables connect the plasma membrane to the condensed chromosomes. The spindle network provides a framework for anterograde and retrograde motors of the kinesin and dynein family to pull the replicated chromosomes apart (115, 172). A group of mitotic kinases (such as AurB, PLK1, and Bub1) and phosphatases work to modulate spindle tension, organize the kinetochore, a specialized multiprotein structure that links the tubulin to the centromere (a unique sequence of DNA recognized by the histone isoform centromere protein A), and checks for connectivity errors (115, 173). Any errors in the formation of this structure lead to mitotic arrest or segregation defects and aneuploidy (142).

Unsurprisingly, the centrosome, astral and spindle microtubules, kinetochores, and centromeres are regulated by O-GlcNAc. OGT strongly localizes to the spindle in both mitosis and meiosis (13, 131), numerous spindle proteins are O-GlcNAcylated (117), and importantly, disruptions in the O-GlcNAc rheostat impact spindle size, shape, and temporal and spatial transitions between mitotic phases (13, 86, 117, 137, 166). Both OGT or OGA overexpression disrupts chromosomal compaction with the length and width of the mitotic chromosomes being larger and less organized (137). Inhibition of OGA with TMG led to more compact spindles than control; furthermore, TMG could partially reverse the disorganized spindle phenotype seen with both OGT and OGA overexpression (137). However, in early prophase, TMG treatment can lead to longer centrosome length (174). Moreover, OGA knockdown caused more compact spindles but also increased the amount of multipolar spindles (86) (Fig. 6). Looking at individual O-GlcNAcylated proteins at the spindle provides some clues into how dynamic O-GlcNAcylation of spindle proteins control spindle formation and function.

MYPT1 is O-GlcNAcylated and interacts with and targets OGT and/or PP1 to potential spindle substrates (169, 174). MYPT1 has several mitotic O-GlcNAcylation sites, which when elevated impair CDK1 phosphorylation of MYPT1; however, when CDK1-phosphorylated MYPT1 interacts with PLK1, reducing PLK1 activity and disrupting spindle organization is (174, 175). Thus, MYPT1 might target OGT, OGA, and PP1 to PLK1 to fine-tune PLK1 activity at the spindle.

Another example of a dynamically O-GlcNAcylated spindle protein is the centrosomal protein nuclear mitotic apparatus protein (NuMA). NuMA is required to maintain spindle shape and organize the spindle pole (176). Although heavily phosphorylated at M phase, NuMA is also O-GlcNAcylated at Ser1844, lowing mitotic phosphorylation. OGT overexpression causes NuMA mislocalization (117). However, NuMA O-GlcNAcylation is required to recruit Galectin-3 to the spindle pole, and alanine mutation of S1844 renders NuMA unable to recruit Galectin-3 resulting in spindle pole disruption (175). Here, O-GlcNAcylation is critical for Galectin-3 recruitment to the spindle, but OGA removal of NuMA O-GlcNAcylation might antagonize OGT activity to regulate NuMA localization and in turn Galectin-3 location.

O-GlcNAc dynamics on specific proteins, the on and off rate of the modification, appear to organize spindle architecture. Yet, the problem is when all the different ways to dynamically process O-GlcNAc at the spindle are added up for individual proteins, the infinite number of protein interactions and PTM cycling renders understanding how O-GlcNAc controls spindle architecture extremely difficult. Does each site on the myriad of O-GlcNAcylated spindle proteins act like a large synchronized group of on/off switches or as a switch that requires additional input? As simple example of this type of additional input switch would be a vending machine. When you put your money into the machine to get a snack, you do not get the snack immediately. The machine now is primed to give you a snack but nothing will happen until you choose chips or pretzels. Once you hit the chip button, then the machine processes your order and provides the chips. Cycling of O-GlcNAc can work in a similar manner. At the spindle, OGT modifies a protein and the resulting O-GlcNAc modified protein interacts with a new subset of proteins, but this new protein complex is only primed for a specific function. Nothing happens at the spindle until OGA removes the O-GlcNAc. The removal allows different proteins to interact with the protein complex driving a functional change at the spindle. Likely, dynamic O-GlcNAcylation at the spindle is a combination of simple on/off switches and more complex switches. Together, these switches can drive spindle formation and function.

## Conclusion

The investment to grow and divide is a consequential decision for the cell that can result in normal growth and cell division or errors leading to apoptosis or cancer. Thus, the cell needs to be in perfect harmony with the environment to grow and divide correctly. The O-GlcNAc rheostat is the precise instrument to integrate all environmental signals to move the cell cycle forward. Thus, as a field, we need to combine multiple experimental approaches to gain a greater understanding of how O-GlcNAc influences cell growth. Novel chemical biology approaches, such as using O-GlcNAc crosslinking agents (177) to identify O-GlcNAc–interacting proteins, would allow mechanistic interrogation of the MCM complex at S phase. Turbo-ID–based O-GlcNAc proximity ligation tools (178) combined with mass spectrometry could quarry O-GlcNAc changes between metaphase to anaphase at the spindle. Combining novel chemical biology techniques with proteomics, O-GlcNAcomics, and metabolomics would integrate changes in O-GlcNAcylation, HBP flux, and cell cycle progression going forward.

## Conflict of interest

The authors declare that they have no conflicts of interest with the contents of this article.
